# Management of Coronary Artery Diseases in Systemic Vasculitides: Complications and Strategies

**DOI:** 10.3390/medicina60101574

**Published:** 2024-09-25

**Authors:** Russka Shumnalieva, Niya Mileva, Ivan Padjen, Periklis Siliogkas, Lyubomir Chervenkov, Konstantina Bakopoulou, Issa El Kaouri, Anna Vasilska, Dimitrina Miteva, Dobrin Vassilev, Tsvetelina Velikova

**Affiliations:** 1 Department of Rheumatology, Clinic of Rheumatology, University Hospital St. Ivan Rilski, Urvich Str. 13, 1612 Sofia, Bulgaria; rshumnalieva@yahoo.com; 2 Faculty of Medicine, Medical University of Sofia, Urvich Str. 13, 1612 Sofia, Bulgaria; 3 Medical Faculty, Sofia University, St. Kliment Ohridski, 1 Kozyak Str., 1407 Sofia, Bulgaria; d.georgieva@biofac.uni-sofia.bg; 4 Cardiology Department, SHATC Medica Cor, Riga Str. 35, 7013 Ruse, Bulgaria; nmileva91@gmail.com; 5 Division of Clinical Immunology and Rheumatology, Department of Internal Medicine, School of Medicine, University of Zagreb, University Hospital Centre Zagreb, Kispaticeva 12, 10000 Zagreb, Croatia; ivan_padjen@yahoo.ca; 6 General Hospital of Athens Korgialeneio—Benakeio Hellenic Red Cross, Athanasaki 11, 11526 Athens, Greece; periaek1@gmail.com; 7 Department of Diagnostic Imaging, Medical University Plovdiv, Bul. Vasil Aprilov 15A, 4000 Plovdiv, Bulgaria; lyubo.ch@gmail.com (L.C.); anna.95@abv.bg (A.V.); 8 Research Complex for Translational Neuroscience, Medical University of Plovdiv, Bul. Vasil Aprilov 15A, 4002 Plovdiv, Bulgaria; 9 Faculty of Medicine, Medical University Sofia, Boulevard ‘Akademik Ivan Evstratiev Geshov’ 15, 1431 Sofia, Bulgaria; 104170@students.mu-sofia.bg (K.B.); 104166@students.mu-sofia.bg (I.E.K.); 10 Department of Genetics, Faculty of Biology, Sofia University “St. Kliment Ohridski”, 8 Dragan Tzankov Str., 1164 Sofia, Bulgaria; 11 Ruse University Angel Kanchev, ul. “Studentska” 8, 7017 Ruse, Bulgaria; dobrinv@gmail.com

**Keywords:** systemic vasculitides, coronary artery disease, immunosuppressive therapy, percutaneous coronary intervention, coronary artery bypass grafting, cardiovascular complications, personalized medicine

## Abstract

Coronary artery disease (CAD) presents a significant risk for patients with systemic vasculitides, a group of disorders characterized by the inflammation of blood vessels. In this review, we focus on the pathophysiological mechanisms, complications, and management strategies for CAD in systemic vasculitides. We highlight how the inflammatory processes inherent in vasculitis contribute to accelerated atherosclerosis and myocardial ischemia. Key strategies in managing CAD in this patient population include using medicine treatments to mitigate vascular inflammation while balancing the risk of promoting cardiovascular events and lifestyle modifications. Understanding the nuanced relationship between systemic vasculitides and CAD is crucial for improving patient outcomes and guiding therapeutic approaches.

## 1. Introduction

Systemic vasculitides represent a group of rare autoimmune disorders characterized by inflammation of the wall of blood vessels of various sizes, from small to large [1]. Cardiovascular involvement substantially contributes to the morbidity and mortality of those affected [1,2] and is often an underrecognized aspect of systemic vasculitides [2]. Although cardiac manifestations in these patients are relatively rare in clinical practice, affecting fewer than 10% of individuals [3], their presence is typically associated with a poorer prognosis. Among their multiple complications, coronary artery disease (CAD) emerges as a significant challenge even in the modern medical landscape. This underscores the critical importance of early detection and timely intervention [4]. Notably, Takayasu arteritis (TA), Kawasaki disease (KD) and eosinophilic granulomatosis with polyangiitis (EGPA) are the forms of systemic vasculitis most frequently linked to heart disease [3].

Furthermore, cardiac involvement is commonly observed in patients who are negative for anti-neutrophil cytoplasmic antibody (ANCA), highlighting the necessity of considering cardiac involvement even in the absence of conventional markers [2]. Research shows that primary systemic vasculitides are a steadily more prevalent contributor to cardiovascular burden among younger patients. Morbidity and mortality in this age group are progressively associated with accelerated atherosclerosis, ischemic heart disease (IHD), venous thromboembolism, and inflammatory/structural myo-pericardial changes [1]. The advent of modern diagnostic possibilities, such as transthoracic echocardiography, cardiac magnetic resonance imaging (MRI), and computed tomography (CT) coronary angiography, has significantly improved the accuracy of diagnosing these conditions. The advanced imaging modalities have not only enhanced diagnostic precision but have also improved the prognosis and informed the management strategies for patients with systemic vasculitides [5].

Understanding the nuanced relationship between systemic vasculitides and CAD is crucial for improving patient outcomes and guiding therapeutic approaches. In this review, we focus on the pathophysiological mechanisms of vascular involvement in specific systemic vasculitides while discussing their clinical manifestations and exploring the complications with which they are associated, thus emphasizing the importance of timely intervention. Moreover, by examining the existing management options, we will provide a clear overview of the current therapeutic approaches and the promising future advances. Advances in understanding disease mechanisms and treatment responses are paving the way for improved patient outcomes.

## 2. Search Strategy

The literature search for this review was conducted using the PubMed, Scopus, and Web of Science databases, covering articles published from January 1960 to July 2024. The search focused on peer-reviewed clinical studies/original articles, reviews, and guidelines for managing CAD in systemic vasculitides. Both Medical Subject Headings (MeSH) and relevant free-text terms were used: “systemic vasculitides”, “coronary artery disease”, “management,” “complications”, and “immunosuppressive therapy.” Boolean operators were used as follows: (“Coronary Artery Disease” OR “CAD” OR “coronary heart disease” OR “CHD”) AND (“Systemic Vasculitides” OR “vasculitis” OR “ANCA-associated vasculitis” OR “giant cell arteritis” OR “polyarteritis nodosa”) AND (“Management” OR “treatment” OR “therapy” OR “strategies”) AND (“Complications” OR “adverse outcomes” OR “risk factors”). We utilized MeSH terms for PubMed and “All Fields” for broader database searches.

**Figure 1:**
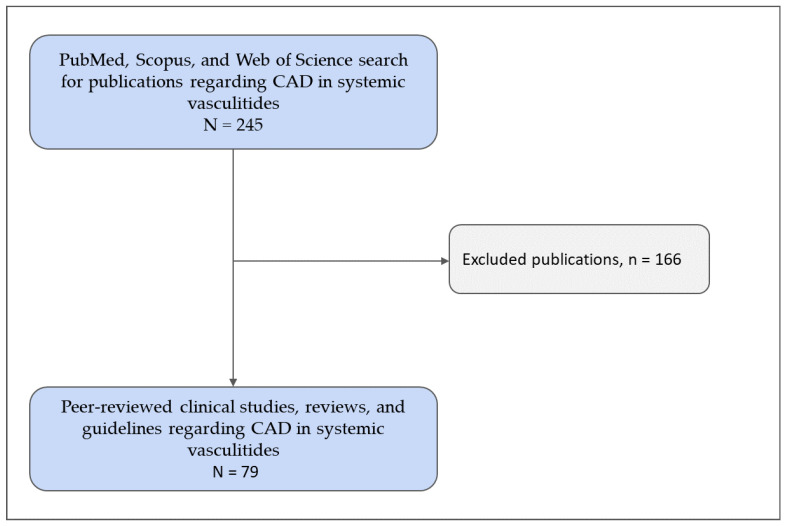
Flowchart of the study inclusion process. CAD—coronary artery disease. Copyright: the authors.

A total of 245 papers were identified, of which 79 met the inclusion criteria, focusing on the clinical management and complications of CAD in patients with systemic vasculitides (Figure 1).

## 3. Pathophysiology of Coronary Artery Diseases in Systemic Vasculitides

### 3.1. Mechanisms of Vascular Inflammation and Damage

Although systemic vasculitides represent a group of pathophysiologically heterogeneous entities, their common denominator is inflammation of the blood vessel wall [6]. Inflammation disrupts the vessel wall structure and its integrity, leading to consequences such as blood vessel stenosis, the formation of arterial aneurysms, or even pseudoaneurysms with vessel wall rupture, as well as in situ thrombosis and distal thromboembolism [6]. End-tissue ischemia is the main hemodynamic consequence related to the territory of the affected blood vessel [6,7]. However, hemorrhages due to vessel rupture or even organ rupture may be observed as well.

Coronary artery vasculitis may be especially challenging given the potentially detrimental consequences of ischemia of both epicardial and non-epicardial coronary arteries. Chronic inflammation may lead to necrosis and scarring of the myocardial tissue. Even in the absence of ongoing inflammation, previous coronary vasculitis is associated with accelerated atherosclerosis and an increased risk of “classical” atherosclerotic CAD [8].

### 3.2. Specific Vasculitides Associated with Coronary Artery Disease

Data on the coronary-specific aspects of the most common vasculitides affecting the coronary arteries are lacking. The knowledge of the pathophysiological aspects of systemic vasculitides affecting the coronary arteries stems mainly from studies assessing the non-coronary arteries.

#### 3.2.1. Giant Cell Arteritis

The inflammatory process of Giant cell arteritis (GCA) seems to be initiated in the adventitia, where dendritic cells become activated by an unknown trigger in a genetically predisposed individual. This, in turn, leads to the activation of CD4+ lymphocytes and their polarization into Th1 and Th17 cells [9]. These cells produce interferon-gamma and interleukin-17, respectively, and are responsible for the migration and accumulation of other cell types in all vessel wall layers. These other cell types include CD8+ lymphocytes, monocytes differentiating into macrophages and giant multinuclear cells, and vascular smooth muscle cells that migrate to the intima, becoming myofibroblasts supporting further vascular stenosis [9].

Interleukin-6 (IL-6), one of the main proteins implicated in the acute phase reaction, has been recognized as one of the main therapeutic targets in GCA and large vessel vasculitis in general. In preclinical models, it has demonstrated its effect in promoting the differentiation of pathogenic Th17 cells from naive T cells. In contrast, IL-6 has been shown to inhibit the development of regulatory T cells [10].

Furthermore, IL-6 has been implicated as a molecule promoting the transendothelial migration of leukocytes via adhesion molecules such as VCAM-1 and ICAM-1 [11].

On the other hand, a recent study on 28 GCA patients has revealed a potential disconnection between the pronounced systemic inflammatory role of IL-6 in GCA and its potentially questionable role in the vessel wall. In this ex vivo study on temporal artery biopsies, intriguingly, IL-6 did not demonstrate an effect on cell migration or cytoskeletal rearrangement. This finding may explain ongoing arterial inflammation in a subset of GCA patients that have achieved reasonable control of the acute phase response using IL-6 inhibitory drugs [12].

#### 3.2.2. Takayasu Arteritis

The prevailing pathogenetic concept of TA is that an unknown stimulus leads to the expression of heat shock protein-65 (HSP-65) in the vessel wall. HSP-65 induces the Major Histocompatibility Complex chain-related A, which is then recognized by T-lymphocytes. This, in turn, drives further inflammation, the migration of monocytes, macrophages, and smooth muscle cells, and intimal proliferation. B-cells also support the inflammatory process by producing anti-aorta, anti-endothelial, anti-cardiolipin, and anti-annexin-V antibodies [13,14].

The inflammatory process of TA seems to start in the inner layers of the blood vessel. In the early stages, inflammation resembles the granulomatous inflammation of GCA (with multinuclear giant cells), whereas histological features are less specific at its later stages [15]. The production of TNF-alpha and interferon-gamma by activated T-cells supports granuloma formation. However, NK and Th17 cells also contribute to the process. At an advanced disease stage, fibrosis is driven by matrix metalloproteinases and TNF-alpha. IL-6 also plays an essential role in supporting vessel wall inflammation and systemic inflammation [15].

#### 3.2.3. Kawasaki Disease

The general understanding is that KD occurs after a child has been exposed to an infectious agent, after which a cascade of events occurs, leading to the hyperactivation of the innate and adaptive immune system. Damage-associated molecular patterns (DAMPs) produced as a consequence of cell death and oxidative stress affect various cells, including leukocytes, platelets, smooth muscle cells, and endothelial cells. Neutrophils and monocytes migrate to the vessel wall and become responsible for the development of coronary arterial vasculitis [16].

In addition to coronary artery vasculitis, the inflammatory cellular infiltrate may not be confined to the blood vessels, yet it can infiltrate the myocardium, causing myocarditis. Interestingly, myocarditis has been described even without the presence of vasculitis. Although myocarditis in the context of KD is usually deemed to be transient, in a subset of patients, it can lead to fatal arrhythmias and myocardial fibrosis [17].

#### 3.2.4. ANCA-Associated Vasculitides

ANCA-associated vasculitides (AAV) are a group of small-vessel vasculitis that are not mediated by immune complexes. Three entities are included in the group: granulomatosis with polyangiitis (GPA), microscopic polyangiitis (MPA), and EGPA [18]. AAV develops due to a loss of tolerance to the main proteins involved in the pathogenesis of the disease—myeloperoxidase (MPO) and proteinase 3 (PR3), both located in the neutrophils. ANCA plays a vital role by activating neutrophils and allowing the release of MPO/PR3 in the microvasculature to be presented by antigen-presenting cells to T-lymphocytes and effector T-cells that mediate further injury. Endothelial and tissue injury spreads beyond the microvasculature, leading to tissue destruction, irreversible damage, and fibrosis [18].

It is worth noting that, among the AAV, EGPA is more frequently associated with cardiac involvement. In addition to small-vessel vasculitis, direct eosinophilic infiltration of the heart tissues (myocardium, pericardium and the endocardium, including the valves) seems to play a role in the pathogenesis of heart disease in EGPA. Abundant eosinophil cells infiltrating the affected tissue exert a variety of cytotoxic and pro-inflammatory effects leading to endocarditis, myocardial dysfunction, and/or pericardial effusion with pericarditis [19]. Furthermore, approximately 50% of the deaths in EGPA are related to cardiac disease and occur within the first few months since diagnosis. However, Dalia et al. discussed that many EGPA patients may not even have cardiac signs or ECG abnormalities [20]. This contrasts with other AAV (ANCA-associated vasculitides) where cardiac involvement is less frequent.

## 4. Clinical Presentation and Diagnosis

### 4.1. Symptoms of Coronary Artery Involvement in Vasculitides

Vasculitides refer to a group of disorders characterized by the inflammation of blood vessels, which can lead to vessel wall damage and subsequent dysfunction of the affected organs. When vasculitis involves the coronary arteries, it can result in significant cardiovascular complications. As the coronary arteries supply blood to the heart, any compromise in their function can lead to severe cardiac conditions [21]. First, we will cover the general symptoms of coronary artery involvement. Coronary artery involvement in vasculitides can present with various symptoms, often mirroring those seen in other forms of CAD. The severity and nature of these symptoms can vary depending on the extent of inflammation and the specific type of vasculitis involved. Common symptoms include chest pain (angina), shortness of breath, fatigue, palpitations, and syncope [21].

KD primarily affects children and is characterized by inflammation of the coronary arteries. Symptoms include fever, rash, conjunctivitis, mucosal changes, and lymphadenopathy. Clinical manifestations may include myocarditis and arteritis, resulting in fibrinoid necrosis of the internal elastic lamina and the subsequent formation of coronary aneurysms in up to one-third of untreated patients [22]. Cerebral aneurysms are less frequent in 1–2% of patients. Monocytes, neutrophils, and macrophages appear to be involved in the pathogenesis of these vascular lesions. Resulting from these inflammatory processes, an inappropriate healing response may also cause coronary stenosis. Otherwise, typical complications associated with the presence of the aneurysms include thrombus formation, causing embolism and peripheral occlusion and rupture. In severe cases, coronary artery aneurysms can form in the coronary arteries, leading to the potential rupture or thrombosis, which can cause myocardial infarction [22].

TA primarily affects young women and involves large vessels, including the aorta and its major branches. Coronary artery involvement can lead to arm or leg claudication, diminished pulses, chest pain, and systemic symptoms: fever, night sweats, weight loss, and fatigue [23]. Angina pectoris may occur following coronary artery ostial stenosis from aortitis or coronary arteritis in 10–45% of the cases. They may have severe clinical sequelae, even though regression upon immunosuppression is possible.

Polyarteritis nodosa (PAN) involves medium-sized arteries and can affect various organs, including the heart. Symptoms related to coronary artery involvement include chest pain due to coronary artery inflammation, damage, or microaneurysms. Coronary involvement may manifest as stenosis, occlusion, aneurysm, or dissection [23]. In a recent review of cases, a total of 34 patients with an average age of 41 years were identified from 32 publications. The male sex is more frequent, and coronary disease was the first manifestation of PAN in ¾. The clinical course of the disease was, in general, very severe, with cases of death from cardiac arrest, pulmonary edema with alveolar hemorrhage, or multiple intracranial hemorrhages after thrombolytic therapy. The formation of immune complexes due to a virus infection (hepatitis B or C) and hairy cell leukemia is thought to mediate the inflammatory reaction, which most often leads to media thickening and stenosis rather than aneurysm formation [24].

Additionally, heart failure symptoms (i.e., dyspnoea, swelling in the legs, ankles, and feet, fatigue, weakness, reduced ability for exercise, pulmonary edema, chest pain, fainting) and arrhythmias (e.g., atrial fibrillation) result from myocardial damage. Hypertension due to renal arteritis and cardiac disease is present in 10–30% of cases [25]. New onset hypertension in a patient with systemic symptoms such as fever, weight loss, and joint pain is a clue of PAN. Other symptoms include deep skin inflammation or progression to infarction and gangrene (30–50%), neuropathy (mononeuritis multiplex in 20–50%), and mesenteric vasculitis.

GCA commonly affects older adults and involves large- and medium-sized arteries, including the aorta and branches. Symptoms related to coronary artery involvement can include jaw claudication, temporal headaches, scalp tenderness, vision problems, and chest pain in cases where the aorta and coronary arteries are involved [25].

MPA and GPA are small-vessel vasculitides that can also involve the coronary arteries, leading to chest pain, pulmonary symptoms (i.e., cough, hemoptysis, and shortness of breath), and renal symptoms (i.e., hematuria and proteinuria, indicating kidney involvement, which can have secondary effects on cardiovascular health) [26]. EGPA primarily affects small-to-medium-sized vessels and is associated with asthma and eosinophilia. Coronary artery involvement can present with chest pain, severe and often difficult-to-control asthma, congestive heart failure, and peripheral neuropathy (i.e., numbness, tingling, and weakness in the limbs [27].

In summary, coronary artery involvement can be identified due to coronary arteritis that causes thromboses, dissections, stenosis, and possible myocardial infarction/ischemia. Coronary involvement was documented in 50% of PAN. It is also widespread in KD (20% of untreated cases) and can be diagnosed via byechocardiography, cardiac computed tomography, cardiac magnetic resonance (CMR), or invasive coronary angiography [28,29].

**Figure 2:**
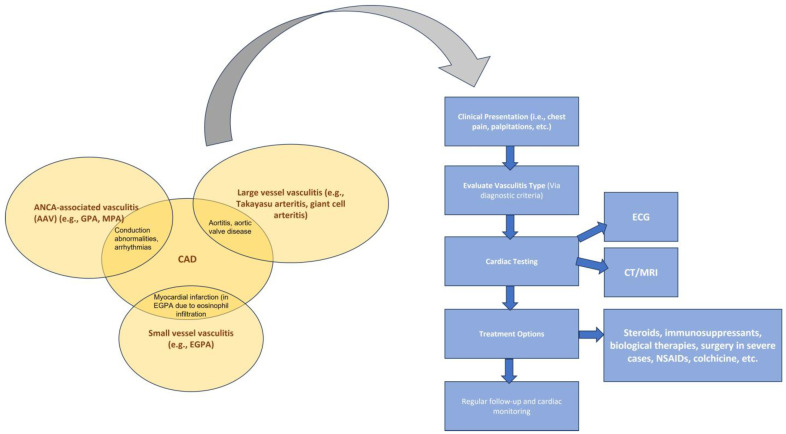
Overlapping cardiac clinical signs of vasculitides and a stepwise clinical approach. Copyright: the authors.

In Figure 2, we present the overlapping clinical features of vasculitides regarding CAD and a stepwise clinical approach.

### 4.2. Diagnostic Imaging and Tests

#### 4.2.1. Transthoracic Echocardiography

Transthoracic echocardiography is usually the initial diagnostic method used to assess the heart and its associated structures in the context of suspected or confirmed vasculitis. The method allows for a comprehensive evaluation of myocardial function and left ventricular ejection fraction, and thus, it enables the detection of possible myocardial ischemia [30]. Due to its noninvasive nature, rapidity, affordability, and safety, echocardiography can be performed repeatedly throughout the course of the disease. However, this technique has several limitations, including poor visualization of the distal coronary artery segments and the physical characteristics of the patient and the necessity for an experienced operator [31].

#### 4.2.2. Coronary Angiography

Invasive coronary angiography is the current gold standard for CAD evaluation. Furthermore, in addition to its diagnostic capabilities, this approach also offers potential for therapeutic intervention [31]. Also, intravascular imaging by means of intravascular ultrasound or optical cocherence tomography can be performed as a supplementary diagnostic tool, enabling optimal visualization and differentiation of the vessel wall layers [32]. The primary limitation of this method is its invasiveness, which may be associated with procedural complications. The method includes the application of X-ray and iodine contrast media, which may be associated with further patient risk.

The role of coronary computed tomographic angiography has been expanding in recent years, largely due to its numerous advantages over other diagnostic methods. CCTA can assess the dimensions and configuration of the arterial wall and lumen, enable plaque characterization and visualize calcifications and thrombi, and evaluate the presence, precise location, and characteristics of aneurysmal dilations or stenotic regions [33]. This method is noninvasive, which gives it a safer risk profile than conventional coronary angiography (CCA). Furthermore, CCTA can perform better in cases of aneurysms or thrombosis, where the size of the lumen may appear normal in other modalities [33]. The advent of the CT technology in recent years led to a reduction in the artifacts related to a high heart rate, thereby facilitating enhanced visualization of the distal branches of the coronary vessels and enabling the reconstruction of intricate anatomical structures in a three-dimensional model [31]. Some potential limitations of CCTA include administering contrast media, using ionizing radiation, and needing a well-trained imaging specialist [34].

#### 4.2.3. Magnetic Resonance Angiography

Magnetic resonance angiography (MRA) is a noninvasive, non-ionizing method for assessing vascular structures, with specific value in examining children and pregnant women [31]. Some studies have reported that cardiac MRA has a lower sensitivity of 93% for detecting coronary vasculitis compared with CCTA, which has a sensitivity of nearly 100%. However, a whole heart cardiac MR can provide a comprehensive evaluation of the myocardial structure, which is crucial for the diagnosis, prognosis, and treatment of these patients [33]. Limitations of the method include its high cost, the need for specific software, technical equipment, and a well-trained and experienced imaging specialist, and contraindications such as claustrophobia and metal implants.

**Figure 3:**
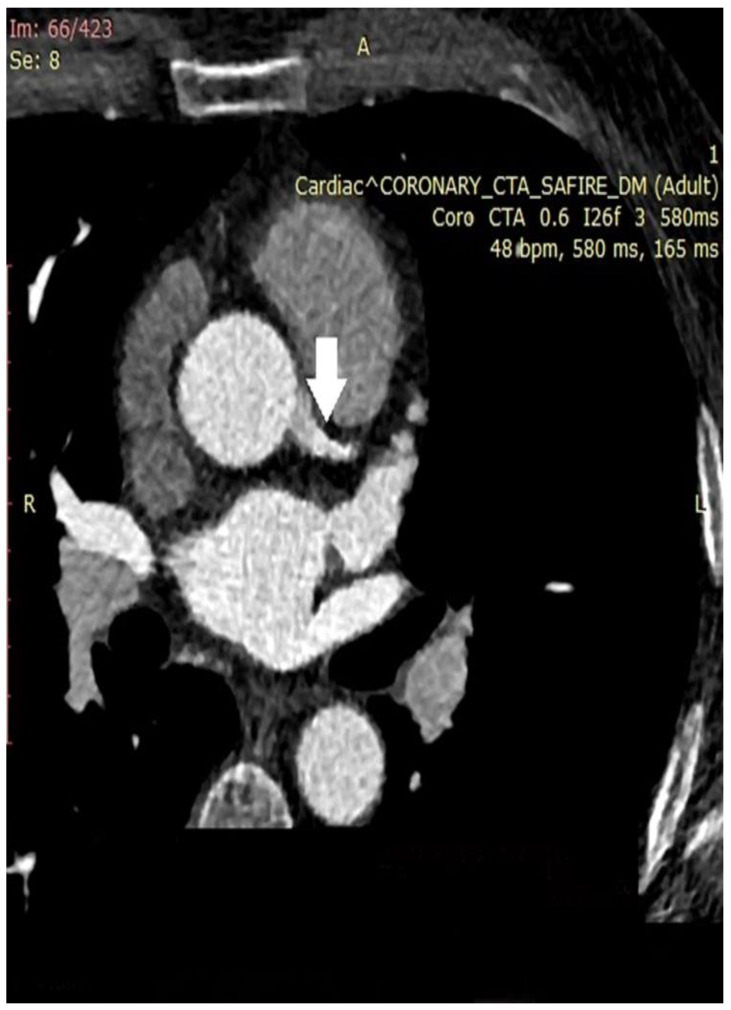
Coronary computed tomographic angiography (CCTA), axial view, and maximum intensity projection (MIP) at the level of the ascending aorta and pulmonary veins. The place of origin of the left main coronary artery is presented (white arrow). A – orientation marker indicating the position of the abdomen; R – orientation marker indicating the right side of the patient. Copyright: the authors.

**Figure 4:**
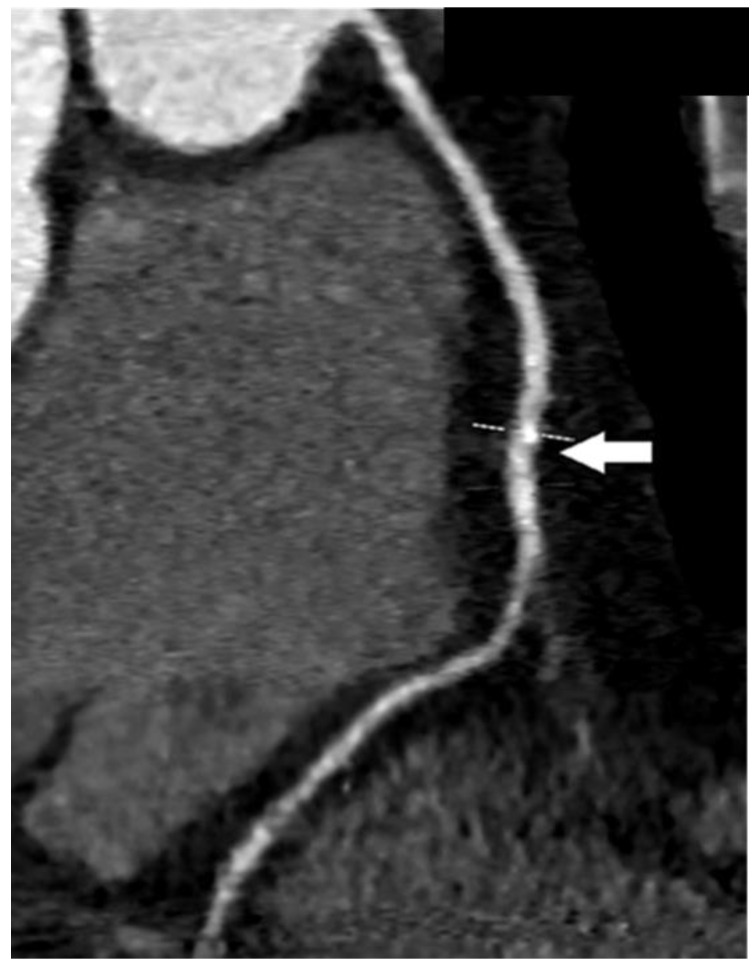
Coronary computed tomographic angiography (CCTA), oblique view, maximum intensity projection (MIP). The right coronary artery is presented with the normal course and lumen width. The white arrow shows a non-obstructive calcification of the artery wall. Copyright: the authors.

**Figure 5:**
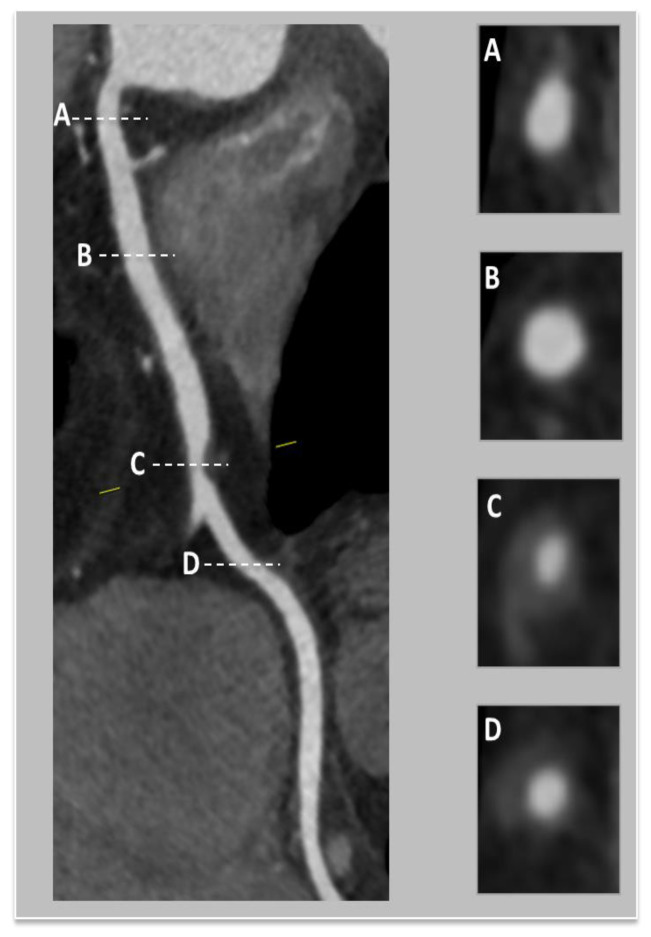
Coronary computed tomographic angiography (CCTA), oblique view, maximum intensity projection (MIP), and cross-sections at different levels of the right coronary artery (RCA). A) Cross-sectional view at the level of ostial RCA with visualization of fibro-lipid plaque and mild stenosis; B) Cross-sectional view at the level of proximal RCA—no evidence of plaque; C) Cross-sectional of the level of mid-RCA—presenting lipid low-attenuation atherosclerotic plaque, causing a significant 50–60% stenosis; D) Cross-sectional view at the level of mid-RCA—noevidence of plaque. Copyright: the authors.

**Figure 6:**
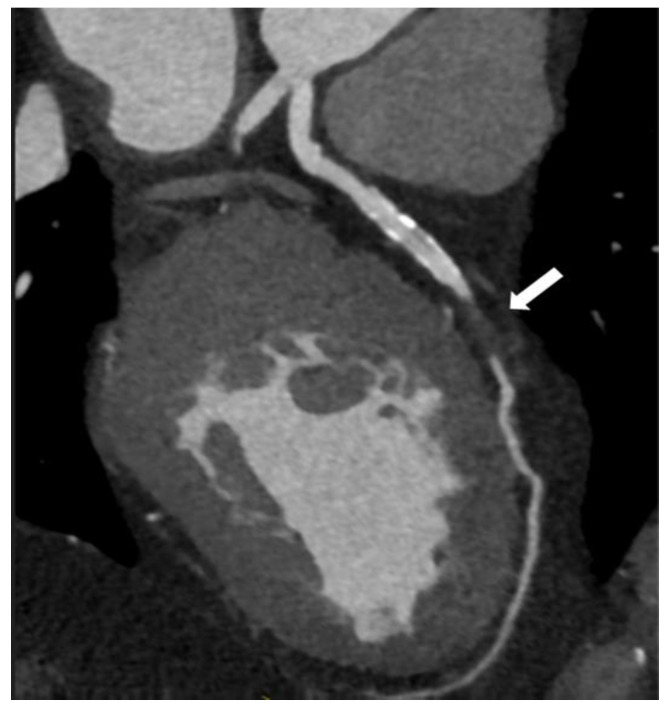
Coronary computed tomographic angiography (CCTA), oblique view, and maximum intensity projection (MIP) present the left anterior descending artery with a total occlusion in the mid-segment of the vessel (white arrow). Copyright: the authors.

In the next figures, we present our experience in the diagnosis of these cardiovascular complications by means of imaging techniques (Figure 3, Figure 4, Figure 5 and Figure 6).

A summary of diagnostic tools for CAD in systemic vasculitides is presented in Table 1.

### 4.3. Differential Diagnosis of CAD in Systemic Vasculitides

The differential diagnoses include other causes of large vessel vasculitis such as inflammatory aortitis (e.g., syphilis, tuberculosis, chronic periaortitis, lupus, rheumatoid arthritis, spondyloarthropathies), developmental abnormalities (coarctation of the aorta and Marfan syndrome), and other aortic pathologies such as ergotism and neurofibromatosis.

Differential diagnoses that can be considered coronary artery affection in systemic vasculitis can include heart palpitations, heart arrhythmias, myocarditis, endocarditis, pericarditis, valvular manifestations, heart failure, aortic dissection, deep vein thrombosis, embolism, hypertension, cerebral stroke, transient ischemic attack, kidney disease (cardiorenal syndrome), peripheral artery disease, antiphospholipid syndrome, lung issues such as shortness of breath, bleeding, coughing, gastrointestinal tract manifestations such as diarrhea, vomiting, hematemesis, stomach pain, chest pain, and neurological conditions presenting with fainting/syncope, dizziness, weakness, and tingling.

We also propose a table that outlines how different manifestations impact the risk of CAD in systemic vasculitides [35] (Table 2).

## 5. Complications of Coronary Artery Diseases in Systemic Vasculitides

Cardiac involvement in systemic vasculitides may be associated with adverse effects such as arrhythmias, myocardial infarction, and even sudden cardiac death [36]. Arrhythmias and conduction abnormalities are essential manifestations of cardiac involvement in systemic vasculitides and can have a severe impact on morbidity and mortality in these patients. The most common arrhythmias include but are not limited to premature supra- and ventricular beats, tachyarrhythmias, and atrial fibrillation [37].

In addition, conduction disturbances such as AV block are another common manifestation of systematic vasculitides. The underlying pathophysiological mechanisms of arrhythmias are complex and multifactorial. However, myocardial fibrosis is considered to be the main mechanism of cardiac involvement, usually as a consequence of an inflammatory process or obstructive CAD [38].

Several possible mechanisms for the association between vasculitis and arrhythmias have been suggested. Firstly, vessel wall inflammation may increase arterial stiffening, impairing the peripheral blood flow and leading to end-organ ischemia. On the other hand, coronary artery damage in systemic vasculitides may be associated with myocardial ischemia and may, therefore, be complicated by myocardial infarction and even sudden cardiac death [39].

There are several cases in the literature describing patients with KD complicated by acute myocardial infarction. Even though data on histopathology reports fail to prove a process of accelerated atherosclerosis in this group of patients, cases of acute myocardial infarction have been published not only in adults but also in children [40,41].

Although much rarer, TA is another systemic vasculitis that an acute myocardial infarction can complicate due to the process of accelerated atherosclerosis [42]. Microvascular dysfunction is another possible pathophysiological mechanism that has been recognized as a potential cause of myocardial ischemia in patients with vasculitis [43].

Coronary microvascular dysfunction may be endothelial-dependent and endothelial-independent based on the specific subset of abnormalities in the microcirculation. The alteration of microvascular function is a pathology that requires specific diagnostic methods and may be easily overlooked. Hence, specific attention should be paid to patients with symptoms of myocardial ischemia and non-obstructive coronary arteries [44].

Many cardiac manifestations may not be clinically evident, especially in the early stages of involvement (Table 3). Therefore, cardiologists should systematically assess cardiac function by applying a multi-modality imaging approach.

**Figure 7:**
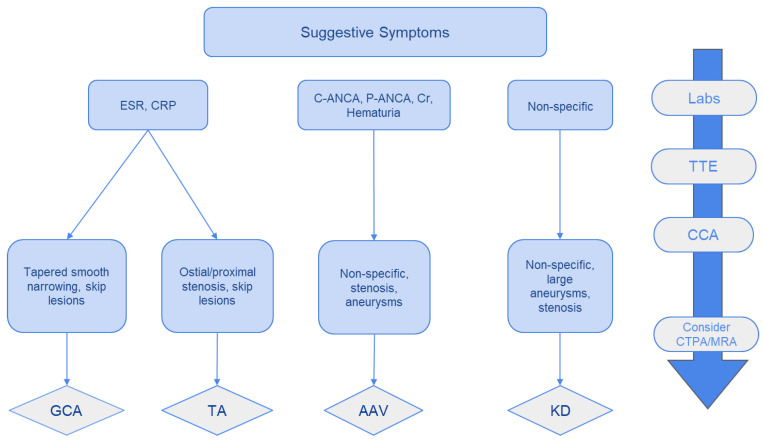
A schematic approach in the diagnosis of coronary vasculitides with CAD. TTE: Transthoracic echocardiography; CCA: Conventional coronary angiography +/− therapeutic intervention; CCTA: coronary computed tomographic angiography; MRA: magnetic resonance angiography. Copyright: the authors.

We propose an algorithm for diagnosing systemic vasculitides presenting with CAD involvement based on laboratory, clinical, and imaging data (Figure 7).

## 6. Management Strategies for CAD in Systemic Vasculitides

### 6.1. Medical Management

Managing cardiovascular manifestations in systemic vasculitides is complex and usually includes glucocorticosteroids (GCS) and immunosuppressants. While GCS are mainly used in the active phase to suppress the disease activity, immunosuppressants are used as steroid-sparing drugs for remission induction and the suppression of disease progression. Novel biological treatment includes the use of TNF-alfa inhibitors, IL-6 inhibitors, and monoclonal antibodies against CD20 [52].

GCS is the first-line treatment in GCA and TA with any manifestations, including CAD. Their usage in a dose ranging from 0.5 to 1 mg/kg is the initial treatment choice for controlling the inflammation. Together with immunosuppressants and heart-failure treatment, GCS improve heart function [53]. In GCA, the use of GCS is crucial for the induction of remission and the prevention of blindness in this subset of patients.

Depending on the severity of clinical symptoms, cardiac involvement in systemic necrotizing vasculitides (SNV), including PAN, GPA, EGPA, and MPA, could be treated symptomatically or by immunosuppression with GCS or cyclophosphamide. GCS are combined with immunosuppressive agents like cyclophosphamide for remission induction in cases of severe, life-threatening manifestations. In the case of GPA, Rituximab could be used both for induction as well as for maintaining remission [54,55,56].

In Behcet’s disease, the use of GCS is controversial as it has been shown that GCS increase the risk of CV mortality [57]. Cyclophosphamide is used as a first-line treatment for remission induction for severe vascular involvement, and azathioprine or mycophenolate mofetil are recommended for maintaining remission in these patients [58,59].

KD is primarily treated with acetylsalicylic acid (ASA) and intravenous immunoglobulin (IVIG). The use of GCS in the acute phase of KD, especially in high-risk patients, leads to improved CAD, clinical symptoms, and laboratory markers for inflammation. According to the literature, using GCS in combination with the standard treatment of KD should be considered in all children, including IVIG resistant- and high-risk patients [60].

Similarly, multiple GWAS aim to reveal KD patients with an increased risk of coronary involvement and IVIG resistance. The results of such studies involving different susceptibility genes have demonstrated a role for calcineurin inhibitors in treating acute KD [61].

Combination therapy with IVIG and corticosteroids in KD patients demonstrated superior efficacy in limiting the occurrence of coronary complications. The response to treatment was found to be directly related to the initial TTE evaluation and the levels of albumin, sodium, hemoglobin, and procalcitonin [62].

### 6.2. Interventional and Surgical Approaches for CAD in Systemic Vasculitides

Percutaneous coronary intervention (PCI) is the predominant method of revascularization in patients with obstructive CAD. However, when it comes to patients with systemic vasculitides undergoing coronary revascularization, most of the reported data come from small studies and case reports [63]. Although some data have been published on the outcomes of PCI in patients with systemic vasculitides, these are from single-center studies with relatively small sample sizes and are usually underpowered to detect statistically significant results [64].

A retrospective study evaluating patients with AAV in the Danish population shows that this subset of patients is at increased risk for coronary revascularization and has a higher associated risk of adverse cardiovascular events [65]. However, the percutaneous revascularization led to a reduction in cardiovascular events over a 3-year follow-up period. Although patients with systemic vasculitides represent a specific subset of patients with advanced progression of CAD, the published data suggest a favorable effect of coronary revascularization. Even after successful PCI or surgical revascularization, vasculitis patients may remain at an increased risk for future adverse ischemic events and the progression of CAD, which is often more advanced than in patients without vasculitis [66]. Currently, there are no officially accepted recommendations regarding revascularization strategies for obstructive coronary disease in these patients. An observational study assessing ninety patients with TA and coronary stenosis reports that in 39 patients, a conservative approach was applied, whereas 51 patients underwent revascularization (28 patients with PCI and 23 with surgical revascularization). The results showed that there was no significant difference in cardiovascular death between the groups with conservative treatment and revascularization. Moreover, cardiovascular mortality was similar in the coronary artery bypass grafting (CABG) and PCI groups, but the restenosis rate was higher in the percutaneous intervention group [67]. In the specific subset of patients with CAD and systemic vasculitis, the decision on the treatment strategy and the revascularization approach should be made after careful consideration and on an individual basis based on the patient’s specific clinical characteristics, the type of vasculitis, and the severity of CAD.

## 7. Emerging Therapies and Future Directions

### 7.1. Advances in Targeted Biological Therapies

For TA, tocilizumab, an anti-IL6 targeting molecule, when used in patients with coronary involvement, reduced the number of active coronary lesions and vessel wall thickening, restoring the function of the coronary artery while simultaneously improving the levels of circulating inflammatory markers (CRP, ESR) and the Kerr score [31].

Tocilizumab in GCA demonstrated decreased dosage requirements for steroids, extended the interval between flare-ups, and reduced the overall frequency of disease flare-ups [68]. A potential pitfall of IL-6 inhibition is the blockade of CRP synthesis. This could potentially mean that ongoing inflammation is concealed by tocilizumab, thus making CRP an ineffective marker for disease monitoring.

Another novel therapeutic approach involves blocking IL-12 and IL-23 pathways by directing a monoclonal antibody against their shared P40 subunit. In this way, ustekinumab inhibits the polarization of Th1 cells towards IFN-γ production while suppressing IL-17 secretion by Th17 cells. Ustekinumab achieved a notable reduction in steroid requirements (from 20 mg to 5 mg) and facilitated complete steroid cessation in ¼ of the patients after approximately one year of treatment for systemic vasculitides [69].

Rituximab is directed against the CD20 molecule on the surface of B cells, leading to their depletion. Standard therapy for AAV includes the combination of a steroid with CYC, AZP, MTX, MMF, or rituximab. However, the significant adverse effects of CYC call for a more precise and less deleterious therapeutic effect. Regarding this aspect, multiple trials (e.g., RITUXVAS) have concluded that rituximab is at least not inferior to cyclophosphamide when used for induction and/or remission for AAV [70].

### 7.2. Role of Personalized Medicine

Personalized medicine attempts to use targeted therapies curated specifically for each patient who has failed to respond to conventional therapeutic options.

As suggested by Yap et al. [71], an example of personalized medicine in the treatment of AAV would entail treating patients according to the type of ANCA present and would abolish the standard classification of EGPA, GPA, or MPA. Another proposed mechanism involves cytotoxic T cells coupled to an autoantibody receptor that binds antibodies, targeting and thus killing PR3- or MPO-expressing B cells in AAV patients.

### 7.3. Potential for Novel Treatment Modalities

Despite the beneficial effect of rituximab in the treatment of GCA and TA being undeniable, it is also accompanied by significant disadvantages, including hypogammaglobulinemia, latent hepatitis B infection reactivation, neutropenia, and immunogenic reactions (due to its chimeric structure). A potential solution to these challenges comes from the emergence of obinutuzumab and ofatumumab, fully humanized antibodies targeting the CD-20 molecule [69].

A very interesting finding in biopsies of the temporal artery from GCA patients revealed the elevated expression of endothelin-1 [72,73]. Therefore, it appears likely that ET-1 inhibition through the employment of existing receptor antagonists could have a central role in the therapy of GCA.

T cell dysregulation has been suspected to contribute to the pathogenesis of AAV following the granulomatous lesions found on kidney and lung biopsies of AAV patients. Therefore, future interventions have shifted towards blocking antigen-presenting B cells from activating T cells using abatacept. The degree to which abatacept can accomplish this should be determined by the ongoing multicenter, randomized, placebo-controlled ABROGATE trial (Abatacept for the Treatment of Relapsing, Non-Severe, Granulomatosis With Polyangiitis) [74].

Alemtuzumab, an anti-CD52 monoclonal antibody, is also being studied for AAV due to its depleting effect on T and B cell populations [75,76,77]. Another novel targeting strategy includes using belimumab to inhibit the maturation of B cells by interfering with their activating factor (BAFF). The BREVAS study (Belimumab in Remission of Vasculitis) aimed to compare belimumab with azathioprine for the maintenance of remission in GPA and microscopic polyangiitis [78].

Further research into the pathogenesis of AAV implicates the inappropriate activation of the alternative complement pathway as a potential target. Avacopan, a C5a receptor inhibitor, is currently considered for its capacity to replace glucocorticoids in the induction and maintenance therapeutic regimens of AAV [79]. A combination of low-dose glucocorticoids and avacopan, avacopan with cyclophosphamide or rituximab induction, or high-dose glucocorticoids was administered to sixty-seven patients with ANCA-associated vasculitis in a randomized trial. At 12 weeks, 86% of the avacopan/glucocorticoid patients and 81% of the avacopan-alone group achieved the primary end-point of the treatment response, which is a 50% reduction from baseline in the Birmingham Vasculitis Activity Score. In contrast, 70% of the glucocorticoid group achieved this goal. All groups had decreases in markers of inflammation and kidney damage, although avacopan caused a faster and more significant reduction. In the high-glucocorticoid group, serious side effects such psychiatric disorders and newly diagnosed diabetes mellitus were more prevalent [79]. These encouraging findings imply that new targeted therapeutics may be able to induce glucocorticoid-free remission in ANCA-associated vasculitis.

This will be investigated further in the bigger, longer-running phase 3 ADVOCATE experiment (A Phase 3 Clinical experiment of CCX168 [Avacopan] in Patients With ANCA-Associated Vasculitis) [80].

Advancements have also been made in treating EGPA, an AAV phenotype characterized by profound eosinophilia. It has been hypothesized that suppressing IL-5 levels with mepolizumab can lead to remission, deplete eosinophils, and allow for steroid dose reduction. In total, 136 patients with refractory or relapsing EGPA were randomly assigned to receive monthly mepolizumab or a placebo in a recent randomized, double-blinded trial [81].

The patients in the mepolizumab arm remained in remission for much longer after 52 weeks; 28% of them remained in remission for more than 24 weeks, while only 3% of patients in the placebo group did the same [81]. The placebo group experienced more serious side effects, while mepolizumab was well tolerated.

## 8. Conclusions

The management of CAD in patients with systemic vasculitides requires a comprehensive approach that addresses both the underlying inflammatory disease and traditional cardiovascular risk factors. The early detection and management of these complications in systemic vasculitides is of utmost importance. A nuanced understanding of the pathogenesis of CAD in this context can inform treatment strategies, optimize outcomes, and minimize complications. Future research should continue to explore targeted therapies and personalized treatment plans to enhance care for these complex patients.

## Figures and Tables

**Table 1:** Diagnostic modalities for CAD and their characteristics: capabilities, advantages, and disadvantages.

Diagnostic Modality	Diagnostic Capabilities	Advantages	Disadvantages
Transthoracic echocardiography	Assessment of myocardial kinetics and presence of ischemia.	Noninvasive Rapid Affordable Safe—Can be performed repeatedly	The necessity of an experienced operator Dependent on a good acoustic window
Coronary angiography	Detailed visualization of the coronary artery tree and stenosis quantification.	Potential for therapeutic intervention	Invasive Severe complications Intravenous contrast media used Radiation exposure
Coronary computed tomographic angiography (CCTA)	Assess the dimensions and configuration of the arterial wall and lumen Plaque characterization Evaluating the presence, location, and characteristics of aneurysm and stenotic regions Reconstruction of intricate anatomical structures in a three-dimensional model	Noninvasive Greater specificity in cases of aneurysms and thrombosis Suitable for follow-up	Expensive Involves ionizing radiation Requires intravenous contrast media Dependent on an imaging specialist
MR angiography	Comprehensive evaluation of the myocardial structure and tissue characterization	Noninvasive Non-ionizing—Useful for the examination of children and pregnant women	Expensive Need for specific software Need for well-trained staff Contraindications such as claustrophobia and metal implants

**Table 2:** The association between different disease manifestations and CAD risk in systemic vasculitides.

Organ/System Involvement	Vasculitides	Association with CAD Risk	Pathophysiological Mechanism
Kidney	GPA, MPA, PAN	Higher Risk	Chronic inflammation and hypertension due to kidney damage accelerate atherosclerosis.
Lung	EGPA, GPA	Higher Risk	Chronic inflammation and hypoxia contribute to increased cardiovascular strain.
Skin	Henoch-Schönlein Purpura, Cutaneous Vasculitis	Lower Risk	Typically, localized inflammation with minimal systemic cardiovascular effects.
Neurological	AAV (e.g., GPA, EGPA)	Unclear	Vasculitic neuropathies may not directly increase CAD risk, but systemic inflammation can contribute indirectly.
Gastrointestinal	PAN, EGPA	Lower Risk	Gastrointestinal involvement has less of a direct impact on CAD, but systemic inflammation may still increase overall cardiovascular risk.

Abbreviations: GPA—granulomatosis with polyangiitis; MPA—microscopic polyangiitis; PAN—polyarteritis nodosa; EGPA—eosinophilic granulomatosis with polyangiitis; AAV—anti-neutrophil cytoplasmic antibodies (ANCA)-associated vasculitides.

**Table 3:** Cardiovascular manifestations and complications in different types of systemic vasculitides.

Disease Entity	Age of Onset (Female-to-Male Ratio)	Type of Vasculitis	Histological Finding	Cardiovascular Manifestations	Coronary Artery Disease	References
Giant cell arteritis	Over 50 Mostly between 70 and 80	large vessel	Classical–transmural inflammation with lymphocytes and macrophages arranged in concentric rings surrounding the external and internal elastic lamina and associated with intimal hyperplasia and the loss of elastic fibers ± giant cells; ±necrosis, granulomas; thrombosis	aortic involvement—dissection or aneurysm peripheral vascular diseases venous thromboembolism stroke myocarditis, pericarditis	Possible association with CAD in patients with vasculitis and no identifiable risk factors for CAD	Hernández-Rodríguez et al., 2016 [45]
Takayasu arteritis	18 ÷ 40 (F:M = 9:1)	large vessel	Active, chronic, or healed lesions; Granulomatous vasculitis, mixed inflammatory infiltrate, including giant cells, presence of necrosis, disruption of the elastic lamina, and features of recanalization	90% aortic involvement a. subclavia involvement—stenosis carotid arteries; renal arteries, pulmonary arteries involvement valvular involvement	Limited stenotic arterial wall lesions due to the inflammation-induced intimal proliferation	Vaideeswar et al., 2013 [14]
Kawasaki disease	under 5	*medium-sized arteries*—coronary arteritis	Inflammatory cell infiltrates mainly from monocytes and macrophages	endocarditis myocarditis valvular involvement CAD rare—valvular sequelae	Coronary arteritis with aneurysm or thrombotic closure	Takahashi et al., 2018 [46]
Polyarteritis nodosa (PAN)	Mostly between 40 and 60 F:M = 1:2	*medium-sized arteries*	Early lesions—fibrinoid necrosis with thickening and infiltration of the vessel wall, neutrophils, lymphocytes and eosinophils, leucocytoclasis; thrombi and aneurysmal changes Mature lesions—vessel occlusion secondary to intimal and mural fibrosis; Nerve injury—fiber loss and axonal degeneration.	vasculitis-related cardiomyopathy coronary artery involvement myocarditis myocardial ischemia, infarction	Coronary arteritis coronary artery aneurism rare—coronary dissection	Stanton et al., 2023 [47]
Granulomatosis with polyangiitis	Mostly between 40 and 60 F:M = 1:1	small-to-medium-sized vessels, capillaries, and venules	Ischemic necrosis with a specific “geographical” organization, with the formation of a nonmicrobial neutrophilic abscess (microabscesses) and a polymorphic granuloma containing polymorphonuclear leukocytes, lymphocytes, plasma cells, dendritic cells, eosinophils, and multinucleated giant cells	Rare: pericarditis, pericardial effusion coronary vasculitis coronary artery aneurysms myocarditis endocarditis valvular lesions (aortic valve) conduction system abnormalities (atrial tachycardia, fibrillation, and flutter, AV block)	Coronary vasculitis coronary artery aneurysms	Masiak et al., 2017 [48]
Eosinophilic granulomatosis with polyangiitis (EGPA)	Mostly between 35 and 50 F:M = 1:1	small- and medium-sized vessels	Tissue eosinophilia, necrotizing vasculitis, and eosinophil-rich granulomatous inflammation	myocarditis (eosinophilic) myocarditis with subsequent restrictive or dilated cardiomyopathy heart failure pericarditis complicated with effusion/tamponade tamponade myocardial infarction	Coronary vessels with myocardial infarction and heart failure coronary spasms manifesting as vasospastic angina	Gioffredi et al., 2014 [49]
Microscopic polyangiitis (MPA)	Mostly between 50 and 60 F:M = 1:1.8	small vessel vasculitis	pauci-immune, necrotizing, small vessel vasculitis without clinical or pathological evidence of granulomatous inflammation	Very rare: pericarditis and pericardial effusion/tamponade valvular abnormalities myocarditis left atrial (LA) dilation LV diastolic dysfunction arrhythmia	CAD due to necrosis of the vessel wall with complete heart block	Park et al., 2019 [50]
Behcet disease	Mostly between 20 and 40 F:M = 4.9–0.36	large, medium, or small arteries or veins	Erythema nodosum-like lesion, Septal panniculitis, dermal perivascular lymphocytic infiltrate, widening of the fibrous septa with edema and infiltration of lymphocytes and histiocytes in the subcutaneous fat, lymphocytic vasculitis	cardiovascular association is between 7% and 46% (venous > arterial involvement) superficial vein thrombosis and deep vein thrombosis pericarditis (38%), endocarditis (26%), intracardiac thrombus (19%), and myocardial infarcts ischemic heart disease low ejection fraction	Coronary aneurysms manifesting as acute coronary syndrome coronary obstruction manifesting as angina, acute coronary syndrome, arrhythmia, acute myocardial infarction	Demirkesen et al. 2010 [51]

## Data Availability

Not applicable.
